# CMM-Based Volumetric Assessment Methodology for Polyethylene Tibial Knee Inserts in Total Knee Replacement

**DOI:** 10.1155/2018/9846293

**Published:** 2018-04-10

**Authors:** Wei Jiang, Cuicui Ji, Zhongmin Jin, Yuntian Dai

**Affiliations:** ^1^School of Mechanical and Automobile Engineering, Changzhou Institute of Technology, Changzhou, Jiangsu 213032, China; ^2^School of Mechanical and Electrical Engineering, Hohai University, Changzhou, Jiangsu 213022, China; ^3^Leeds Joint School, Southwest Jiaotong University, Chengdu, Sichuan 611756, China

## Abstract

Total knee replacement is a common surgical procedure in orthopaedics. Accurate volumetric wear assessment of the polyethylene knee inserts has been an essential subject for improving the longevity. A new CMM-based methodology was presented to determine volumetric material loss based on curve surface fitting without prewear data, CAD model, or original design of drawings. Both computational and experimental simulated volume removal tests were run to validate the methodology by comparing with the gravimetric measurements. The volume and linear wear of the tibial inserts were calculated using the presented method based on the coordinates acquired by the CMM. The results indicate that the methodology is adequate for clinically retrieved tibial inserts where no prewear data are provided. This technique can also be used for biotribological study of other polyethylene components, since wear and damage can be assessed visually and volumetrically.

## 1. Introduction

Total knee replacement (TKR) is being widely used as a successful and effective treatment of degenerative knee joint diseases, about 80 percent of which were carried out because of osteoarthritis of the knee, and the number of knee replacement operations is increasing every year worldwide [1]. CoCrMo alloy, ultra-high molecular weight polyethylene (UHMWPE), and more recently, ceramics are used in the prosthesis manufacturing process for reducing the wear and improving the longevity of implants thanks to their significant advantages in terms of low friction coefficient and good antiwear property. However, wear of polyethylene bearing component is a major problem in total knee replacement, and studies have shown that about 16 percent of knees fail due to polyethylene wear [2]. Therefore, accurate wear assessment of the polyethylene knee inserts has been an essential subject for improving the longevity [3]. Wear measurement methodologies become critically important if differentiations with respect to materials and design are sought when geometry change is small, which can consist of both wear and creep. There are many methods of determining the volume loss of polyethylene in the hip, knee, and spine either using contact or noncontact procedures. Volumetric measurements are commonly performed using tactile coordinate measuring machines (CMM) [4–6] or noncontact techniques such as micro X-ray computed tomography (CT) [7] and gravimetric method [8, 9]. CMM has been proved to be an accurate technique for volumetric assessment [10, 11]; however, CMM measurement can induce deformations on polymeric-bearing components due to clamping and probing forces [12]. The aim of this study was to develop a novel methodology based on three-dimensional (3-D) geometry acquired by means of a tactile CMM to determine volumetric material loss of polyethylene tibial knee inserts and validate its effectiveness on the basis of computational and experimental studies of simulated volume removal tests.

## 2. Materials and Methods

**Figure 1:**
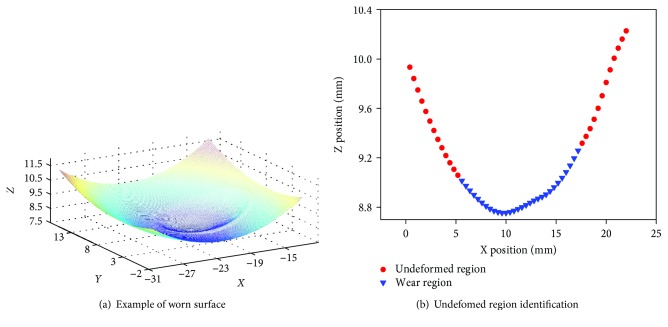
Example of polyethylene worn surface and undeformed region identification.

**Figure 2:**
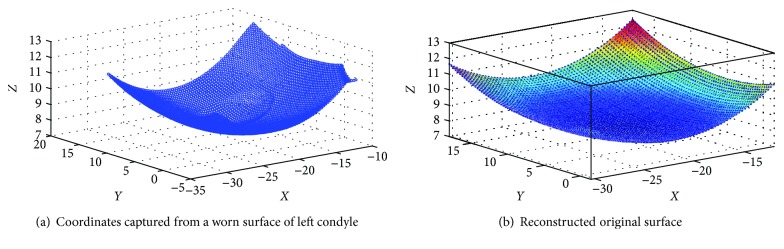
Surface coordinates obtained using CMM and reconstructed original surface for volumetric assessment.

An unworn PFC Sigma tibial knee component (manufactured by DePuy Synthes, UK) was used for the experimental investigations presented in this work. The original surface coordinates of the left condyle were obtained using a coordinate measuring machine (Mitutoyo Legex 322). The coordinates obtained from the unworn tibial knee inserts were considered as the prewear data and used as reference for both computational and experimental simulated volume removal tests, which were used to validate the volumetric assessment methodology by comparing with the prewear data. From the captured 3-D coordinates, a three-dimensional surface was then established. The wear region was determined according to the difference of *Z* value between two adjacent coordinates, and it can be judged to be worn out when the difference is greater than 0.1 mm. It is important to note that some clearly wrong coordinates need to be removed for accurate wear region identification. Thus, the undeformed region was identified automatically using a MATLAB (Version 8.3, Mathworks Inc., USA) program (Figure 1), which was used as a reference for volume loss assessment in the conditions where no prewear data was provided [13, 14]. A 5th-order polynomial curve surface fitting algorithm (1) was used to generate the original 3-D surface based on this undeformed region (Figure 2).

(1) $$\begin{array}{c} f\left(x,y\right)=P00+P10x+P01y+P20x^{2}+P11xy+P02y^{2}+P30x^{3}+P21x^{2}y+P12{xy}^{2}+P03y^{2}+P40x^{4}+P31x^{3}y+P22x^{2}y^{2}+P13{xy}^{3}+P04y^{4}+P50x^{5}+P41x^{4}y+P32x^{3}y^{2}+P23x^{2}y^{3}+P14{xy}^{4}+P05y^{5}, \end{array}$$

where *Pij* are the parameters in polynomial surface fitting algorithm, *i* is the degree in *x*, and *j* is the degree in *y*.

**Figure 3:**
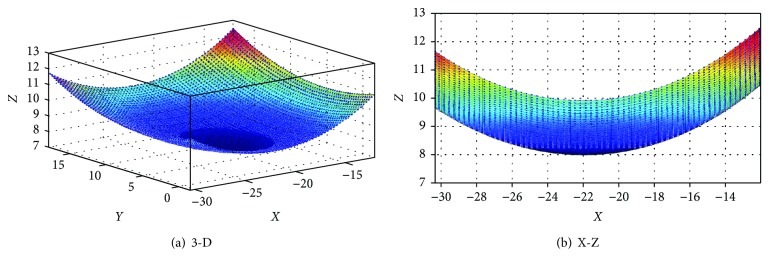
Computational simulated volume removal test (the dark blue area is the worn region).

**Figure 4:**
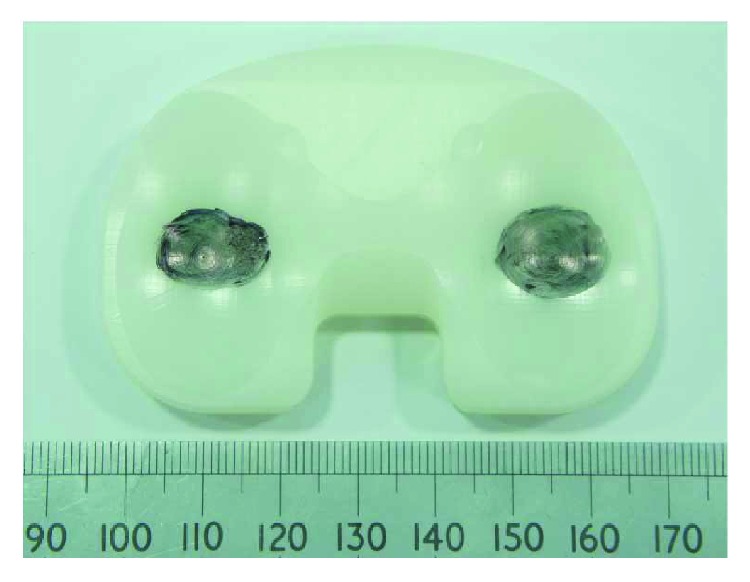
Experimental simulated volume removal test (the black area with the ink painting is the worn region).

Prior to the CMM measurement, the polyethylene inserts were cleaned using detergent water then soaked in 1% Trigene solution (MediChem International Ltd., Seven Oaks, UK) to clean the specimen for 30 minutes to remove contaminants from the surface. Afterwards, the inserts were soaked in isopropanol solution (Fisher Scientific, Loughborough, UK) mixed with water (70% isopropanol: 30% water) and placed in an ultrasonic bath (VWR Labshop, IL, USA) for 10 minutes (IMBE simulator test protocol, Leeds University, UK). Then, the components were stored in the weighing room, which is temperature and humidity controlled (21°C and 40%, resp.) and allowed to stabilize for a period of 48 hours. The change in mass was assessed using the AT 201 balance (Mettler Toledo Inc., Columbus, Ohio, USA), and the volumetric loss was calculated using 2, taking the density of polyethylene as 0.931 g/mm^3^ [5]. For computational simulated volume removal test, the coordinates from the left condyle were used for the development of a computational program to artificially generate different wear areas and depths via a MATLAB program (Figure 3). The wear region of the left condyle ranges from 0.29% to 38.55%, and the maximum wear depth was 0.2 mm. For experimental simulated volume removal test, a 24 mm diameter ball-ended cutter was used to remove physical materials on the left condyle of the tibial knee inserts with maximum wear depths from 0.1 mm to 1 mm (Figure 4). The volume loss of the polyethylene tibial knee inserts was calculated using the presented methodology based on the coordinates captured by the CMM, respectively.

(2) $$\begin{array}{c} Volume\;loss=\frac{weight\;change}{density}. \end{array}$$

## 3. Results and Discussion

**Figure 5:**
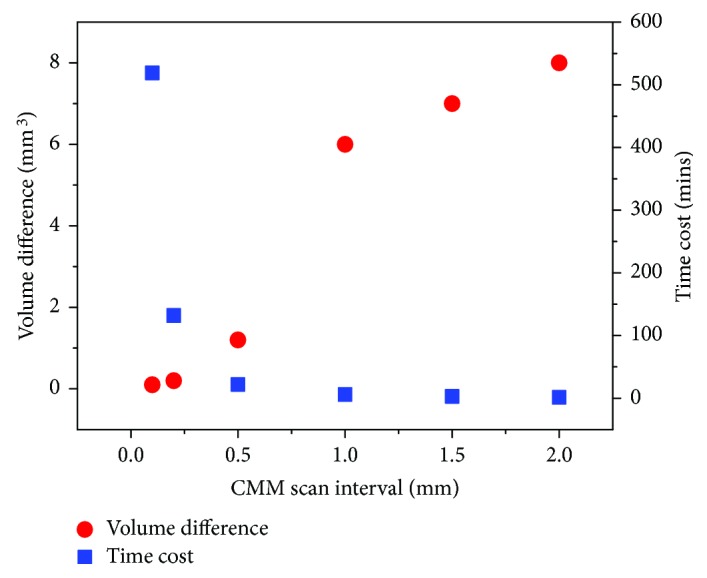
The influence of CMM scan interval on volume difference.

**Figure 6:**
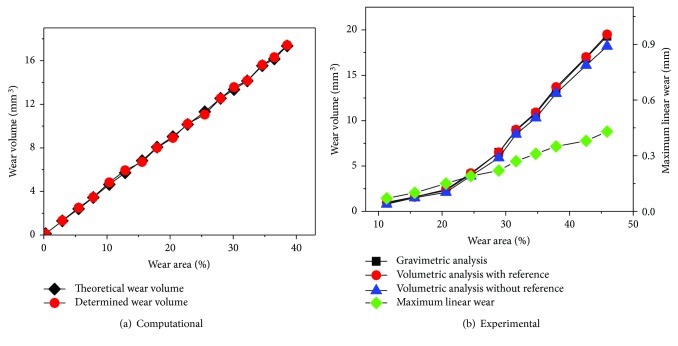
Computational and experimental simulated volume removal test results.

**Figure 7:**
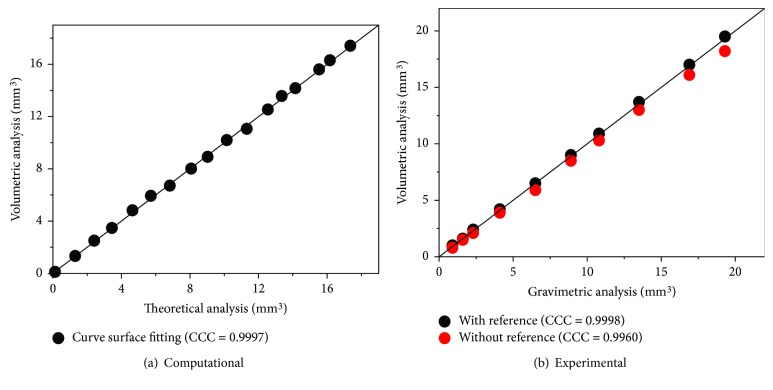
Concordance correlation coefficients results of volumetric assessment methodology based on computational and experimental simulated volume removal tests.

An unworn tibial knee component was used to investigate the influence of the CMM scan interval (0.1 mm, 0.2 mm, 0.5 mm, 1.0 mm, 1.5 mm, and 2.0 mm), and the results were demonstrated in Figure 5, with the increasing interval of the CMM scan, the points measured decreased from 31,133 to 90 and the volume difference increased from 0.1 mm^3^ to 8.1 mm^3^, meanwhile the time taken decreased from 519 minutes to 1.5 minutes. As a result, the scan interval with 0.2 mm was adopted in this study to balance accuracy and time costs (7872 points measured and time taken was 132 minutes). As shown in Figure 6(a), a total of 17 computational wear tests were performed on the left condyle of the tibial knee component to generate different volumes of wear with an increasing wear area. A comparison of theoretical wear volume calculated using coordinates before and after wear test and determined wear volume calculated using surface curve fitting was performed. The simulated volume loss generated using computational model ranges from 0.1 mm^3^ to 17.4 mm^3^, and the determined wear volume was very close (maximum error equal to 0.2 mm^3^) to the theoretical with concordance correlation coefficients (CCC) of 0.9997 (Figure 7(a)). As illustrated in Figure 6(b), the gold standard gravimetric measurement was chosen as a reference for validation of the 3-D curve surface fitting method in physical volume removal tests. The wear volume generated by the ball-ended cutter was gravimetrically measured using the AT 201 balance (Mettler Toledo Inc., Columbus, Ohio, USA) and ranged from 0.9 mm^3^ to 19.3 mm^3^, and the validation results indicated that the methodology is accurate for assessment of wear volume (maximum errors equal to 0.2 mm^3^ and 1.1 mm^3^, resp.), with CCC of 0.9998 and 0.9960 with and without initial surface coordinates, respectively (Figure 7(b)). The corresponding wear volume assessed by the presented methodology ranged from 1.0 mm^3^ to 19.5 mm^3^ and from 0.8 mm^3^ to 18.2 mm^3^, respectively.

Initial geometric measurement of specimens or design drawings would be ideal as a reference for volumetric wear assessment; however, these are not always available [15, 16]. This study presented a CMM-based methodology to determine volumetric material loss based on 3-D curve surface fitting, and the validation results indicated that the methodology is adequate for both laboratory and clinically retrieved tibial knee inserts where no prewear data, CAD models, or original design drawings are available. Further studies indicated that at least 50 percent undeformed region of each condyle was required for accurate volumetric assessment, as proved in physical volume removal tests (Figure 4). With the increase of wear area, the undeformed region will not be enough to reconstruct the initial surface, which will have a great influence on the volumetric assessment. There were some limitations in this study, such as the uncertainty in the actual machining tolerance and plastic deformations generated during physical volume removal tests, which are likely due to the vibration. Furthermore, the clamping and probing forces during the CMM measurement can induce deformations to the polyethylene tibial knee components, which is not visible when comparing the volumetric determination with and without reference; however, this should be taken into account as an uncertainty contribution and needs further studies. However, the general high levels of agreement indicate that this method is appropriate to measure clinically relevant levels of wear.

## 4. Conclusions

This paper presented a coordinate-based volumetric wear assessment methodology for polyethylene tibial knee inserts in total knee replacements. In the cases of no prewear data such as CAD models and design drawings provided, the original condyle surface was generated via a 5th-order polynomial curve surface fitting algorithm based on the unworn coordinates obtained using CMM. The influence of scan interval range from 0.1 mm to 2.0 mm was investigated using an unworn tibial knee component, and the scan interval of 0.2 mm was used in the CMM measurement to balance accuracy and time costs. Both computational and experimental simulated volume removal tests were performed to validate the accuracy of the methodology. For computational simulated volume removal tests, the determined volume loss was very close (maximum error equal to 0.2 mm^3^) to the theoretical with concordance correlation coefficients (CCC) of 0.9997. For experimental simulated volume removal tests, the validation results show slightly deviation but still indicated that the methodology is accurate for wear volume assessment (maximum errors equal to 0.2 mm^3^ and 1.1 mm^3^, resp.), with CCC of 0.9998 and 0.9960 with and without initial surface geometry, respectively. The presented CMM-based methodology can be used for volumetric assessment and can also be applied to the biotribological study of other polyethylene components, since wear and damage can be assessed visually and volumetrically.
